# Tightly linked antagonistic‐effect loci underlie polygenic phenotypic variation in *C. elegans*

**DOI:** 10.1002/evl3.139

**Published:** 2019-09-11

**Authors:** Max R. Bernstein, Stefan Zdraljevic, Erik C. Andersen, Matthew V. Rockman

**Affiliations:** ^1^ Department of Biology and Center for Genomics & Systems Biology New York University New York New York 10003; ^2^ Molecular Biosciences and Interdisciplinary Biological Sciences Program Northwestern University Evanston Illinois 60208

**Keywords:** Complex traits, genetic architecture, linkage

## Abstract

Recent work has provided strong empirical support for the classic polygenic model for trait variation. Population‐based findings suggest that most regions of genome harbor variation affecting most traits. Here, we use the approach of experimental genetics to show that, indeed, most genomic regions carry variants with detectable effects on growth and reproduction in *Caenorhabditis elegans* populations sensitized by nickel stress. Nine of 15 adjacent intervals on the X chromosome, each encompassing ∼0.001 of the genome, have significant effects when tested individually in near‐isogenic lines (NILs). These intervals have effects that are similar in magnitude to those of genome‐wide significant loci that we mapped in a panel of recombinant inbred advanced intercross lines (RIAILs). If NIL‐like effects were randomly distributed across the genome, the RIAILs would exhibit phenotypic variance that far exceeds the observed variance. However, the NIL intervals are arranged in a pattern that significantly reduces phenotypic variance relative to a random arrangement; adjacent intervals antagonize one another, cancelling each other's effects. Contrary to the expectation of small additive effects, our findings point to large‐effect variants whose effects are masked by epistasis or linkage disequilibrium between alleles of opposing effect.

**Impact Summary**

Genetic association studies have suggested that nearly every region of genome harbors variants that affect typical complex traits, providing ample fuel for evolution. In this study, we test these findings using experimental genetics in *C. elegans*. We used high‐throughput phenotyping to measure a genetically complex multivariate quantitative trait. We asked whether 0.001 of the genome is likely to carry alleles that detectably influence the trait. For nine of 15 such intervals, we found that allelic differences between two strains conferred significant phenotypic effects. Surprisingly, these effects were not small. If such effects were present across the genome, the total amount of variation would greatly exceed the variation we observe. Our findings point to large‐effect variants whose effects are masked by epistasis or linkage disequilibrium between alleles of opposing effect.

A detailed understanding of the genetic architecture of complex traits is necessary to address questions about the origin and maintenance of heritable phenotypic variation and the mechanisms of adaptation. Many models explain heritable variation as the result of an equilibrium between the introduction of variation by mutation and its erosion by drift and stabilizing selection. For traits affected by enormous numbers of loci, particularly in species with low effective recombination rates, linkage between variants may also be important. Very tightly linked variants, inherited together, can act as coupling‐phased supergenes that increase phenotypic variance or repulsion‐phased linkage blocks that reduce phenotypic variance. Under stabilizing selection near an optimum, we may expect an excess of the latter case: tightly linked variants with opposite effects. Near a phenotypic optimum, mutations that counteract the effect of linked variants will be favored, and recombination between antagonistic‐effect loci will be disfavored (Fisher 1930; Mather 1943; Lewontin 1964).

Experimental investigation of tightly linked polygenes is challenging. Variants that occur in linkage equilibrium in natural populations are necessarily too weakly linked to provide the relevant long‐term selective effects, rendering association‐type methods ineffectual for this question. Polygenes are expected to have miniscule effects, mandating very high levels of experimental replication. We therefore characterized linked polygene effects directly by using high‐throughput phenotyping and high levels of replication in experimental panels of recombinant *Caenorhabditis elegans*. This species is well suited for this type of study, as it has a short generation time and is naturally inbred (Barriere and Felix 2005; Gray and Cutter 2014; Frezal and Felix 2015). These features allow for relatively quick construction of recombinant panels and high‐throughput assays in 96‐well plates (Andersen et al. 2015). Although many traits in *C. elegans* have a simple genetic basis (e.g., Palopoli et al. 2008; Ghosh et al. 2012; Noble et al. 2015; Zdraljevic et al. 2017), complex traits in *C. elegans* often have polygenic or otherwise complex architectures (Green et al. 2013; Glater et al. 2014; Andersen et al. 2015; Greene et al. 2016; Evans et al. 2018; Noble et al. 2017).

A key feature shaping genetic variation in *C. elegans* is the species’ androdioecious mating system; most individuals are self‐fertile hermaphrodites, incapable of mating with one another, and so most wild individuals are completely homozygous, the product of a predominantly selfing history. Rare males, which arise by X‐chromosome nondisjunction, can mate with hermaphrodites, introducing rare outcrossing events. Overall, the mating system means that dominance is expected to play a negligible role in the patterning of variation, and the low rate of effective recombination contributes to strong linkage disequilibrium and its attendant evolutionary consequences (Cutter et al. 2009; Felix and Braendle 2010; Rockman et al. 2010; Andersen et al. 2012). In short, in this species new mutations rarely experience genetic backgrounds other than those on which they arose, particularly over short genetic distances and short evolutionary timescales.

To characterize the genetic architecture of complex trait variation in *C. elegans*, we used a sensitizing condition, excess of the metal nickel, to expose variation that might not be visible under favorable laboratory conditions, and we measured individual and population growth rates in animals from two recombinant panels: a large set of recombinant inbred advanced intercross lines (RIAILs) from a cross of strains N2 and CB4856 (Andersen et al. 2015), and a collection of near‐isogenic lines (NILs) carrying small regions of CB4856 donor genome on the X chromosome within an otherwise N2 background (Bernstein and Rockman 2016) (Fig. 1). RIAILs leverage genotypic replication across random backgrounds, whereas NILs control for background by holding it constant (Eshed and Zamir 1995; Koumproglou et al. 2002; Keurentjes et al. 2007; Doroszuk et al. 2009; Shao et al. 2010). RIAILs provide an efficient way to survey the whole genome for loci with significant marginal effects across multiple backgrounds, but those multiple backgrounds also contribute phenotypic variation. Thus, when comparing the phenotype distributions for two genotype classes at a given genetic marker, RIAILs have abundant variation within each class due to segregating genetic effects. With NILs, those background effects are eliminated, providing greater power to detect differences between focal genotypes.

**Figure 1:**
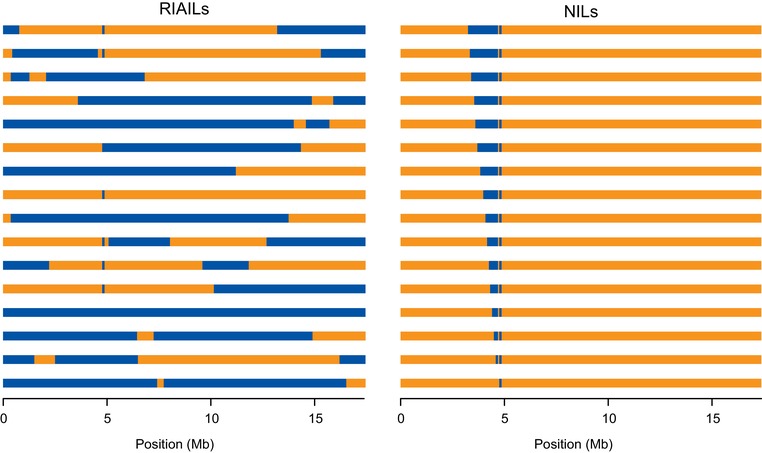
X chromosomes of the two genetic mapping panels. Left: Sixteen of 282 recombinant inbred advanced intercross lines, each homozygous for a unique mosaic of N2 (orange) and CB4856 (blue) genomes. At any specific marker, approximately half the lines are homozygous N2 and the remainder homozygous CB4856. Right: Near isogenic lines derive almost entirely from the N2 background but carry small regions of CB4856 genome within a 1.4 Mb region on the X chromosome. Each CB4856 interval shares a common right end, so that pairs of most‐similar strains differ only by 53–148 kb of genome. In both panels, every strain also carries *qgIR1*, a 110‐kb introgression of CB4856 genome at 4.8 Mb; this introgression carries the ancestral allele of *npr‐1*, where N2 carries a laboratory mutation (see Supporting Information). The introgression is included in the experiment to avoid potentially confounding effects of this large‐effect mutation.

We tested polygeny by estimating the probability that a small piece of genome, 0.1% of the whole, harbors allelic differences that affect growth and reproduction. We tested for antagonism between linked intervals by comparing the effects of the intervals to those expected under alternative arrangements. And we tested for the generality of our findings by making a model with parameters that could jointly explain the observed variation in the NIL and the RIAIL panels.

## Methods

#### Overview

Our experimental goal was to measure quantitative traits at high replication in a large number of genetically characterized inbred strains (Fig. 1). For each assay, we initiated a population with three L4 hermaphrodites and then used a worm sorter to count and measure the progeny of those founders, yielding a multivariate population‐level phenotype that captures aspects of fecundity and growth rate (see Results section and Fig. S1 for details). We then performed statistical tests to evaluate the contribution of genetic variation to the measured phenotypic variation.

#### Strains

We used 282 strains from the Andersen panel of Recombinant Inbred Advanced Intercross Lines (Andersen et al. 2015; Zdraljevic et al. 2017) and 16 strains from the Bernstein panel of Near Isogenic Lines (Bernstein and Rockman 2016). Details of strain construction are provided in the Supporting Information.

#### Growth assays

The RIAILs and the NILs were assayed by worm sorter, as described previously (Andersen et al. 2015; Zdraljevic et al. 2017). The assay conditions are detailed in the Supporting Information, and phenotype is described in detail in the Results section. Sorter data were processed using the R package COPASutils (Shimko and Andersen 2014), which is available at github.com/Andersenlab/easysorter.

For the RIAIL experiments, each of the 282 RIAILs was assayed once. These experiments took place over 10 assay days. For all analyses of RIAIL data, we used as phenotypes the residuals of a multivariate linear regression of raw phenotypes on assay day, modeled as a factor.

Assays of NILs were performed at much higher replication, to allow for well‐powered pairwise comparisons of strains (Fig. S1). This kind of replication is not necessary in the RIAILs because each allele is present in roughly half the RIAIL strains, providing effective replication for the effects of each locus. Prior to each of three NIL assay days, each of the 16 NILs was grown and passaged in five independent replicates for four generations to reduce or eliminate shared transgenerational environmental effects. Then on the assay day, each of the five independent populations of each strain was grown in a well on each of nine to 11 different assay plates, with positions of each NIL randomized across assay days. In all, this amounts to 2312 assays: 3 assay days × 16 strains × 5 passaging replicates × 9–11 growth assay replicates. One strain was assayed on only two of the three assay days, and 20 assay wells were excluded from analysis as outliers, leaving 2293 assays (mean 143.3 per strain). All results are robust to the treatment of outliers.

#### Statistical analyses

We performed all statistical tests and analyses in R (R Core Team 2017). Fixed‐effect multivariate analyses used the R package *car* (Fox and Weisberg 2011), and mixed‐effect models used the package *lme4* (Bates et al. 2015). The raw data for the RIAILs and NILs are provided as Supporting Information Files S1 and S2, and an annotated reproducible pipeline for all analyses is present in Supporting Information File S3.

#### Linkage mapping in RIAILs

To identify regions of genome that harbor allelic differences that affect phenotypes, we performed multivariate marker regression (Knott and Haley 2000) with a forward search strategy (Doerge and Churchill 1996). The model and fitting procedure are described in Supporting Information. Ten of 282 RIAILs had five or fewer progeny per initial worm and were excluded from analysis.

#### Analysis of near‐isogenic lines

We accounted for experimental variation in our measures of NIL demography by treating assay day, assay plate, well position, and passaging replicate as random effects in univariate analyses of each trait. This analysis yielded estimates (Best Linear Unbiased Predictors) for each trait for each of 237 independently passaged replicate populations (3 assay days × 16 strains × 5 passaging replicates, less some missing data). We used this 237‐observation dataset to test whether genotype accounts for phenotypic variation, as described below.

To test for the effect of each interval on the multivariate demography phenotype, we compared two strains at a time. For each pair of strains that differ by a single interval (that is, the top two NILs in Fig. 1, or the second and third NILs, or the third and fourth, etc.), we used a multivariate analysis of variance to ask whether strain identity explained any of the phenotypic variation. We used this same approach to test for a significant difference between the two parental NILs (i.e., the top and bottom NILs in Fig. 1). To estimate *P*‐values for these comparisons, we used permutations, shuffling the strain labels among the observations for the pair of strains in each test. We used these permutations to derive null test statistic distributions for univariate trait comparisons as well.

## Results

### HIGH‐THROUGHPUT MEASUREMENTS OF MULTIVARIATE PHENOTYPES

To characterize the genetic basis for variation in growth and reproduction, we used a previously established pipeline for high‐throughput population phenotyping (Andersen et al. 2015; Zdraljevic et al. 2017). We measured phenotypes for 272 RIAILs grown in liquid cultures containing 350 µM nickel chloride. We chose NiCl_2_ because a preliminary survey of diverse stressors suggested that the left side of the X chromosome, a region for which we had previously generated NILs, carried a quantitative trait locus (QTL) for a nickel‐by‐genotype interaction. After placing three L4 hermaphrodites in each well of a microtiter plate and allowing them to mature and produce progeny over four days, we passed each resulting population through a COPAS BIOSORT large‐particle sorter (Union Biometrica). The sorter counts the number of animals in each well, and for each animal it measures time of flight, which serves as a measure of body length. The result of each assay is thus a histogram of the body lengths observed in the broods of three hermaphrodites at a fixed time (Fig. S1). We divided the body‐length histograms into three bins: less than 90 µm (small), 90–200 µm (medium), and greater than 200 µm (large), and we calculated the proportion of worms from each well in each bin. Under control conditions, these bins correspond to developmental stages L1, L2 + L3, and L4 + adult. These stage assignments are unlikely to hold under nickel stress, but they nevertheless provide a simple way of describing the body‐length distribution. The three body‐length–bin proportions within a well must sum to one, so we can use any two proportions to provide a description of the body‐length distribution of the well. We combined three traits—the proportion of worms in the Small size bin, the proportion of worms in the Medium size bin, and Number of progeny per founding L4—into a three‐dimensional phenotype vector [SMN] for our subsequent genetic analyses. As a shorthand, we refer to this vector as “demography.” This vector is simply a way of summarizing the body‐length histogram in a manner that retains interpretable features of worm biology. We chose a multivariate approach because it improves power in cases where QTL affect combinations of traits (Jiang and Zeng 1995; Korol et al. 1995; Knott and Haley 2000; Stephens 2013), and because it obviates problems with multiple hypothesis testing in the context of high‐content high‐throughput data.

### MULTIPLE QTLs AFFECT REPRODUCTION AND GROWTH IN RIAILS

**Figure 2:**
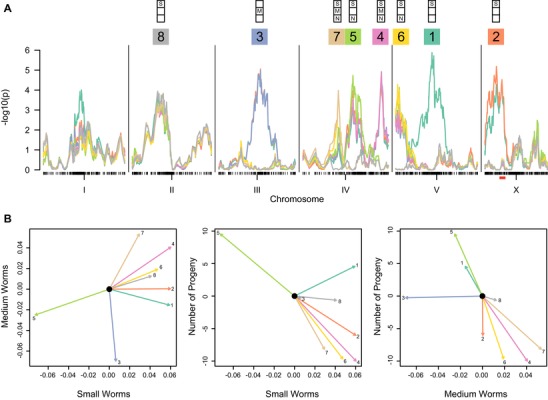
Quantitative trait loci in RIAILs. (**A)** Multivariate QTL scans with a forward search strategy identified an eight‐locus genetic model. Test statistic profiles (−log_10_(p)) for eight sequential scans are plotted in different colors, and the QTL retained from each scan indicated by its number and color. The *x*‐axis represents genetic position. The solid red box below the X chromosome marks the NIL interval dissected later in the paper. Each QTL is annotated according to whether its univariate effect is nominally significant (*P* < 0.05) for small worm proportion (S), medium worm proportion (M), or number of progeny (N). (**B)** Bivariate projections of the QTL effects. Each vector shows the estimated effect of the CB4856 genotype at the indicated QTL, numbered and colored as in panel A.

We performed multivariate QTL mapping to identify regions of the genome that influence demography in the RIAILs. We employed simple multivariate marker regression (Knott and Haley 2000) on the assay‐corrected RIAIL phenotypes, and we used a forward search strategy with a genome‐wide *P* = 0.05 permutation‐based residual empirical threshold (Doerge and Churchill 1996). This approach identified eight significant QTL (Fig. 2A). The effects of the CB4856 allele at each QTL, projected into bivariate space, are plotted in Figure 2B, and the underlying phenotype data are in Figure S2. Many of the QTL influence multiple aspects of demography, although several are restricted to one or a few trait axes. For example, QTL 3 affects the proportion of worms in the medium size class, but it has little effect on progeny number or the proportion of small worms. For each trait, we observe QTL where the CB4856 allele increases the trait value and others that decrease it. For example, QTL 4 and 5, linked on chromosome IV, are nearly collinear in three‐trait space, but with effects in opposite directions. In other words, the parental strains carry mixtures of antagonistic alleles.

We found no evidence for pairwise or higher order epistasis among the detected QTL (*P* = 0.52 and 0.99 for comparisons to a purely additive model), and the eight‐QTL model explains 29% and 16% of the variance in the small and medium size worm proportions, and 8% of the variance in progeny number.

### NEAR ISOGENIC LINES PROVIDE A DIRECT TEST OF A POLYGENIC ARCHITECTURE

One possible genetic model for our traits ascribes the unexplained phenotypic variation to a large number of variants spread across the genome. Under this polygenic model, any region of the genome is likely to harbor phenotypically penetrant variants. We used a panel of 16 Near Isogenic Lines to test 15 consecutive intervals of 53–148 kb (i.e., ∼0.001 of the 100 Mb genome each) spread along a 1.4 Mb region on the X chromosome (Bernstein and Rockman 2016). Among the RIAILs, this region is partly contained within QTL 2 (Fig. 2A), although it does not include the QTL peak (Fig. S3).

The NILs allow for straightforward tests, comparing two strains that differ only in a single interval. These strains enable us to control for loci outside the interval (thereby removing a major source of within‐marker‐class variation), and they expose the variation within the interval that could be masked by tightly linked antagonistic QTL. In total, there are 1838 SNPs and 635 indels in the NIL interval (Thompson et al. 2015). We assayed growth in three independent experiments (Fig. S1). In each of the three experiments, each strain was grown in five independent replicate populations and passaged over several generations, prior to phenotype assays, to reduce shared environmental effects. Each of these passaging replicates was then assigned to a random well position in a 96‐well plate, and that plate layout was replicated across 9–11 plates within that assay day. In total, we analyzed NIL demography in 2293 assays (mean 143.3 per strain). The design allows us to test the effect of genotype while accounting for variation due to experimental factors. Note that our NIL analysis compares two genotypic classes, each measured approximately 143 times, whereas in the RIAIL analysis, each marker genotype class is present in approximately 136 (= 272/2) strains.

**Figure 3:**
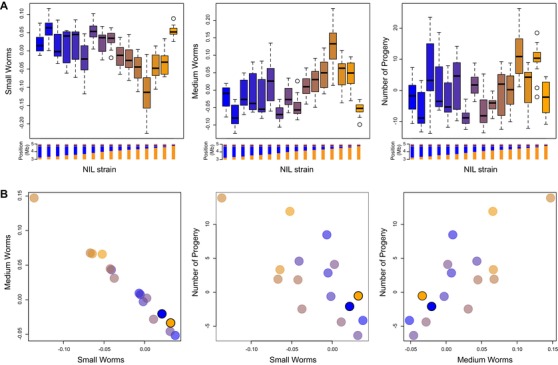
Phenotypes of 16 near isogenic lines. (A) Boxplots show the distribution of trait values for ∼15 replicates of each of the 16 NILs, after accounting for variation due to experimental factors. (B) Multivariate strain phenotypes from a fixed‐effect model, with each NIL colored as in panel A. The parental NILs are highlighted.

To account for variation due to experimental factors, we used univariate mixed‐effect models to extract phenotype values (best linear unbiased predictors) for each of the ∼15 replicate populations of each NIL (3 assay days × 5 passaging replicates, less some missing data), using the entire dataset to account for variation due to assay day, assay plate, and well position. We then applied a fixed‐effect multivariate model to estimate each strain's demography phenotype. As shown in Figure 3, the NILs vary in demography. Most of the variation is confined to a subspace, as the proportions of small‐ and medium‐sized worms are highly negatively correlated among the NILs (Fig. S4). The parental NILs, one entirely N2 and the other entirely CB4856 within the NIL region, differ from one another slightly but significantly (*P* = 0.001). The differences between the parental NILs are limited to the size‐class distribution, as the number of progeny is indistinguishable (*P* = 0.79; Fig. 3).

### MULTIPLE QTLs ARE FOUND WITHIN THE NIL INTERVAL

**Figure 4:**
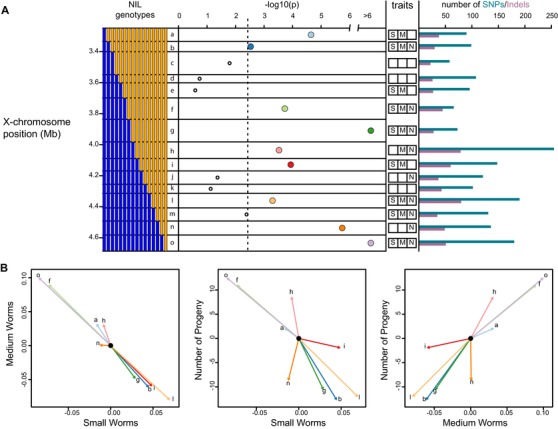
NILs reveal antagonistic QTL. (**A)** The genotypes of the 16 NILs, at left, define 15 intervals (a‐o). By comparing strains that differ only in a single interval, we tested for the effect of the interval on demography, with the results plotted as −log_10_ of the *P*‐value. The dashed line indicates the Bonferroni‐adjusted significance threshold, *P* = 0.05/15. The trait column indicates which intervals have nominally significant (*P* < 0.05) univariate effects on each of the three traits (small, medium, and number). The bar plot at right shows the number of SNPs and indels in each interval. Each significant point is colored to facilitate comparison with panel B. (**B)** Estimated effects of the CB4856 genotype for each significant NIL interval.

We tested whether the demography phenotype of each strain differed significantly from that of the genetically adjacent strain, thereby testing each of 15 genomic intervals. Twelve of the 15 intervals contained nominally significant QTL, nine at a Bonferroni‐adjusted *P*‐value threshold of 0.003 (Fig. 4A; Table S1).

The estimated effects of each NIL interval are plotted in Figure 4B, and they reveal several striking patterns. First, as in the case of the RIAILs, the effects point in both directions for each trait, indicating that the NIL region harbors a mixture of antagonistic QTL. Adjacent intervals often have effects in opposite directions. For example, intervals *f* and *g* result in an increase and decrease in the number of progeny, cancelling one another's effects. Second, many effect vectors are nearly collinear, consistent with the reduced range of variation in certain axes of phenotypic variation. For example, intervals *f, l*, and *o* have effects that nearly occupy a line in three‐dimensional space, with *l*’s effect in the opposite direction to that of *f* and *o*. Most of the intervals have pleiotropic effects, and some effect directions are absent. For example, no interval simultaneously increases the number of progeny and the proportion of small worms. As in the RIAILs, some of the NIL QTL affect only one or a few of the phenotypic axes. For example, interval *n* acts almost exclusively on progeny number. In general, the orientations of the effects are quite different from those observed for the QTL detected in the RIAILs (Fig. 2B), indicating a substantially different genetic correlation structure in the two experimental panels. Finally, the magnitudes of the effects in the NILs are large, comparable to those detected in the RIAILs. For example, many of the NIL interval effects change the number of progeny per animal by 10 offspring.

We found that 60% of ∼100 kb windows had significant phenotypic effects in this assay (nine out of 15). If we assume that these windows are typical samples of the 100 Mb genome, simple extrapolation implies that N2 and CB4856 differ in about 600 100 kb intervals with significant effects on the phenotype. The large effect sizes in the NILs raise the question of whether a genome full of such effects is consistent with the variation observed from genome‐wide segregation in the RIAILs. We therefore simulated a RIAIL phenotype (number of progeny) by assigning effects to a random 600 of the markers genotyped in the RIAILs, with effects drawn from a normal distribution with the inferred NIL effect‐size mean (−0.1) and variance (8.6). In a million simulated datasets, the simulated RIAIL variance was on average 11 times greater than the observed RIAIL variance, and never as low as the observed variance. Other approaches to simulating the effect sizes, including drawing from a uniform distribution or resampling directly from the NIL effects, yielded similar results (Fig. S5).

The simulations aimed to synthesize the RIAIL and NIL findings, and they reject the simplest such synthesis, wherein the NIL interval effects are simply assigned to random positions in the genome. An alternative model is that the causal variants of opposite sign tend to be tightly linked more than expected under a random distribution of effect signs (i.e., adjacent antagonistic QTL cancel one another). Under such a model, we predict (1) that the sequence of effects along the 15 intervals in the NILs should not be random but instead should roughly alternate in sign, and (2) simulations of coupled canceling QTL should recapitulate the observed RIAIL variance.

**Figure 5:**
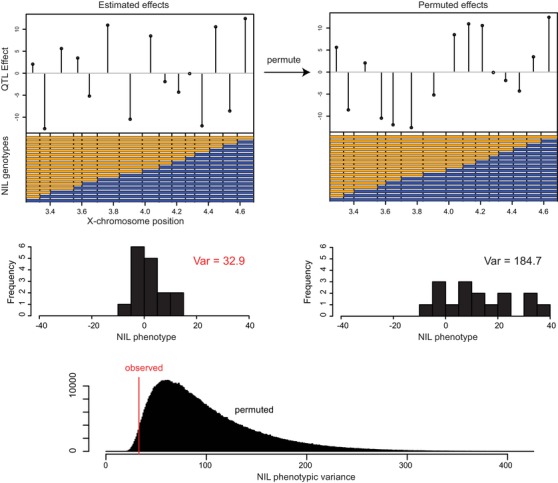
The QTL effects of the NIL intervals increase and decrease the phenotype, number of progeny. The estimated effect of substituting a CB4856 allele (blue) is plotted in the top left panel. The phenotype of the all‐orange N2‐like NIL is zero and the phenotype of the all‐blue NIL is the sum of the plotted effects. The histogram below shows the phenotype distribution of the NILs. To generate a null distribution of phenotypic variance, we permuted the order of the NIL effects. At right, an example of permutation is given. The distribution of phenotypic variance for a million shuffled orders is shown below, with the observed variance indicated by the red line.

To test the first prediction, we asked whether the phenotypic variance among the NILs is smaller than we would observe if the NIL intervals were randomly ordered. We estimated the null distribution of this variance by permuting the genetic effects among the NIL intervals. The null hypothesis of random QTL effect arrangement is rejected with *P* = 0.014 (Fig. 5). That is, the QTLs in the NIL intervals are arranged in a sequence that significantly reduces phenotypic variance relative to a random arrangement.

The phenotypic effect of the 1.4 Mb NIL interval as a whole—the difference between the parental NILs—is a relatively modest 1.6 progeny (Fig. 3). If we assume that the NIL interval is typical, and that there are 100 such intervals across the genome, simulations are consistent with the variance observed among the RIAILs. For example, if we model 100 QTLs with effects drawn from a normal distribution with standard deviation 4.5 (rather than 600 QTLs with standard deviation 8.6), and we assume arbitrarily that the heritability of progeny number in the RIAILs is 0.5, then the mean simulated RIAIL variance matches the observed RIAIL variance, and the general features of the RIAIL QTL distribution are also replicated (Fig. S6) (cf. Visscher and Haley 1996). Notably, this model is only one of an enormous number of possible genetic models that could synthesize the NIL and RIAIL findings, but it demonstrates that additive effects in tight repulsion‐phase linkage could account for the observations. Arbitrary models of suppressive epistasis could also produce these findings.

## Discussion

For more than a century, experimental and theoretical studies have examined the extent to which phenotypes are polygenic. Evidence from experimental studies describing quantitative trait variation suggests that polygeny is the norm (Mackay et al. 2009; Rockman 2012; Boyle et al. 2017). For example, recent analyses of human genetic variation have inferred that the majority of 1 Mb windows harbor variation that affects schizophrenia risk (Loh et al. 2015), and most 100 kb windows affect height (Boyle et al. 2017). These estimates require assumptions about the relationship between allele frequency, effect size, and linkage disequilibrium (reviewed by Yang et al. 2017), and direct assessment of individual polygene effects is difficult in the context of small effects, complex genetic backgrounds, and low minor allele frequencies. Here, we used a classical genetics approach to isolate small genomic intervals and directly assess their effects on complex traits. Our NIL‐based analysis of small genomic region provides a simple and direct validation of polygeny: most intervals carry segregating variation that affects a complex trait. Both of our experimental panels, RIAILs and NILs, revealed that two strains, CB4856 and N2, harbor large numbers of allelic differences that affect demographic traits under stress.

Nine of 15 intervals, each ∼95 kb on average, had significant effects on the phenotypes. The focal region of the X chromosome is not particularly noteworthy with regard to trait variation as a whole in these strains (Andersen et al. 2015; Evans et al. 2018). Its SNP density is similar to the X chromosome arms as a whole, and the X chromosome arms are considerably less SNP‐dense and indel‐dense than the autosome arms (Thompson et al. 2015). Our simple extrapolation to 600 causal intervals genomewide is likely conservative, given the probability that tightly linked variants within the nonsignificant intervals may cancel each other's effects, causing us to miss them, as we observed for our parent NILs. Moreover, our power to detect very small effects remains quite limited. Projecting to the broader *C. elegans* population, our sample of two strains provides a narrow view of phenotypically relevant genetic variation. N2 and CB4856 differ in our 1.4 Mb focal genomic region by 1838 SNPs, while a survey of 249 distinct wild isolates (isotypes) identified more than 8900 single nucleotide variants segregating in the region (Cook et al. 2017, release 20170531; Hahnel et al. 2018).

The genetic variation that we analyze derives from the two most widely studied *C. elegans* isolates. N2 is the canonical reference strain. Isolated from mushroom compost in Bristol, England, in 1951, the strain experienced substantial adaptive evolution in the laboratory prior to its cryopreservation in 1969 (McGrath et al. 2009, 2011; Sterken et al. 2015) (only one variant within the NIL region, in interval *o*, arose in N2 after its isolation; McGrath et al. 2011). CB4856, isolated from a pineapple field in Maui in 1972, was for many years the *C. elegans* isolate most different from N2, and these two strains have been the subject of an enormous number of genetic studies. Both strains have exceptional high‐quality genome assemblies (Consortium 1998; Thompson et al. 2015; Kim et al. 2019; Yoshimura et al. 2019). Population genetic studies have revealed that the similarity of most wild isolates to N2 results from very recent partial selective sweeps and migration events that have homogenized much of the species, while CB4856 retains alleles that were lost in the swept populations (Rockman and Kruglyak 2009; Andersen et al. 2012). Many other additional isolates are now known that retain ancestral variation (Cook et al. 2017).

Our results contribute to a growing consensus that tightly linked antagonistic QTL (whether additive or epistatic) are a common feature of complex‐trait architectures (Steinmetz et al. 2002; Kroymann and Mitchell‐Olds 2005; Shao et al. 2008; Gaertner et al. 2012; Green et al. 2013; Glater et al. 2014; Mackay 2014; Metzger and Wittkopp 2019). Partial selfing may facilitate the evolution of these complexes, and they may contribute to the widely observed pattern of outbreeding depression in the partially selfing *Caenorhabditis* species (Dolgin et al. 2007; Baird and Stonesifer 2012; Gimond et al. 2013; Snoek et al. 2014). Although this pattern may be most common in selfers, it should arise in any species in traits under stabilizing selection with tight linkage, as evidenced by excess repulsion‐phase linkage disequilibrium between coding and *cis*‐regulatory variants in humans (Castel et al. 2018). Moreover, for the pattern that we observe—polygeny and tight linkage of antagonistic effects—to arise by stabilizing selection, some outcrossing and recombination is required; otherwise, antagonistic alleles could sit anywhere in the genome and we would not predict an alternating sequence.

Tight repulsion‐phase linkage disequilibrium provides a simple model for the generation and storage of cryptic genetic variation in natural populations (Mather 1943; Lewontin 1964; Kroymann and Mitchell‐Olds 2005; Hansen 2006; Paaby and Rockman 2014). In Lewontin's (1964) words,

“Despite the very low genetic variance in the tightly linked cases, the opportunity for the manifestation of new genotypes is much greater because gene frequencies are held at intermediate frequencies. The tightly linked genes then have a greater potential to respond to new selective forces because potential genetic variability is maintained in the form of linked complexes.”

On longer timescales, tight linkage among antagonistic‐effect loci may explain the widely observed phenomenon of developmental systems drift (True and Haag 2001), whereby cellular and developmental events are conserved despite extensive functional turnover at the molecular level (Ludwig et al. 2000; Barriere et al. 2012).

Our data show that a simple model of additive effects in repulsion‐phase linkage disequilibrium can account for the mismatch between the amount of variation in RIAILs and the magnitude of differences between nearly identical NILs. However, more complicated models are also possible. In particular, our NIL data do not directly address the possibility that the large effects we observe are normally masked by epistatic interactions among adjacent intervals. If epistasis is systematically suppressive (Hansen et al. 2006; Hansen 2013), then the effects of isolated variants will routinely exceed their effects in their native genetic background. Such a pattern has been observed repeatedly in laboratory mice (Shao et al. 2008; Tyler et al. 2016), and several experiments have pointed to large stores of normally cryptic epistatic variation in *C. elegans* that can be exposed by genetic perturbations (Paaby et al. 2015; Snoek et al. 2017; Sterken et al. 2017). Whether by repulsion‐phase additive effects or tightly linked epistatic variants, *C. elegans* harbors a store of variation beyond that exposed by ordinary segregation.

Associate Editor: S. Wright

## Supporting information

Supporting Information

Supporting Information

Supporting Information

**Figure S1**. Experimental workflow for NILs.
**Figure S2**. Assay‐adjusted multivariate phenotypes for 272 RIAILs.
**Figure S3**. The NIL interval falls on the shoulder of QTL 2.
**Figure S4**. A total of 237 estimated phenotypes for the 16 Near Isogenic Lines.
**Figure S5**. The NIL QTL imply excessive phenotypic variance for the RIAILs under a null model of randomly distributed QTL.
**Figure S6**. A representative simulation of 100 QTLs with effects drawn from a normal distribution with standard deviation 4.5. The phenotypes of 272 RIAILs were simulated by assigning the QTL to 100 markers in the RIAIL genotype data, calculating the expected phenotypes, then adding an equivalent amount of random variation (i.e., expected broad sense heritability is 0.5).
**Table S1**. Permutation‐based NIL p‐values.
