# Supplementary material for: Tightly linked antagonistic‐effect loci underlie polygenic phenotypic variation in C. elegans
Source: Evol Lett. 2019 Sep 11;3(5):462–73. doi: 10.1002/evl3.139 (PMC6791183; doi:10.1002/evl3.139)
Supplement: Supplementary file 4 — Figure S1. Experimental workflow for NILs. Figure S2. Assay‐adjusted multivariate phenotypes for 272 RIAILs. Figure S3. The NIL interval falls on the shoulder of QTL 2. Figure S4. A total of 237 estimated phenotypes for the 16 Near Isogenic Lines. Figure S5. The NIL QTL imply excessive phenotypic variance for the RIAILs under a null model of randomly distributed QTL. Figure S6. A representative simulation of 100 QTLs with effects drawn from a normal distribution with standard deviation 4.5. The phenotypes of 272 RIAILs were simulated by assigning the QTL to 100 markers in the RIAIL genotype data, calculating the expected phenotypes, then adding an equivalent amount of random variation (i.e., expected broad sense heritability is 0.5). Table S1. Permutation‐based NIL p‐values. [file EVL3-3-462-s004.pdf]

Supplementary Information

Strain construction

In the construction of both the RIAIL and NIL panels, the N2-like parental strain carried *qgIR1*, an introgression of 110 kb of CB4856 genome on the X chromosome that includes the *npr-1* locus. This gene carries a laboratory-derived mutation in the N2 strain, and the N2 allele has major effects on a broad range of *C. elegans* phenotypes (McGrath *et al.* 2009; Andersen *et al.* 2014; Sterken *et al.* 2015; Zhao *et al.* 2018). We were concerned that the effects of the N2 allele would swamp or modify the subtle effects of natural variation (*cf.* Stinchcombe *et al.* 2009). The *qgIR1* introgression replaces N2 allele with the ancestral wild-type *npr-1*. Therefore, every NIL and RIAIL carries *qgIR1* and this interval does not contribute to variation within the panels. The RIAIL N2-like parent, QX1430, also carries *ttTi12715*, a transposon insertion in the *peel-1* gene that reduces the paternal-effect incompatibility between it and CB4856 (Andersen *et al.* 2015).

Construction of the NILs involved use of visible marker mutations, *fax-1(gm83)* and *lon-2(e678),* as described in Bernstein and Rockman 2016. The two strains that ultimately gave rise to the NILs, QG613 (*fax-1 qgIR3 qgIR1* X) and QG590 (*lon-2 qgIR1* X), carry background genetic mutations that may contribute to phenotypic variation among the NILs. We whole-genome shotgun sequenced these strains and identified a total of 72 autosomal mutations that distinguish the two strains, including eight predicted nonsynonymous mutations. The cross design used to generate the NILs should exclude the possibility that X-linked mutations present in the parental strains, outside the NIL interval, vary among the NILs. Thus each pair of assayed strains is expected to differ at ~36 sites in addition to those within the focal interval. Although background mutations may contribute to phenotypic variation, they are not expected to generate the variance-reducing pattern we observe (*i.e.,* Figure 5)

Worm Sorter Assays

Prior to assays, strains were passaged on plates for four consecutive generations to reduce any transgenerational effects from starvation or other stresses. Strains were then bleach-synchronized and aliquoted to 96-well growth plates at approximately one embryo/µl in K medium (Boyd *et al.* 2012). Embryos were then allowed to hatch overnight to the L1 larval stage. The following day, hatched L1 animals were fed HB101 bacterial lysate (Pennsylvania State University Shared Fermentation Facility) at a final concentration of 5 mg/ml and allowed to grow to L4s over the course of two days at 20ºC. Three L4 larvae were then sorted using the Union Biometrica Large-Particle flow cytometer (BIOSORT) into experimental plates containing HB101 lysate at 10 mg/ml, K medium, 31.25 µM kanamycin, and 350 µM nickel chloride dissolved in water. The animals were then grown for four days at 20ºC. During this time, the animals matured to adulthood and laid embryos that subsequently hatched and commenced feeding and growing. Prior to measuring the resulting populations' demographic parameters, animals were treated with sodium azide (50 mM) to straighten their bodies for more accurate length measurements.

Linkage mapping

We performed multivariate marker regression (Knott and Haley 2000). The model is **Y** = **XB** + **E**, where **Y** is the 282 x 3 matrix of RIAIL phenotypes, **X** is a 282 x 2 design matrix, with 1s in the first column and marker genotypes (0 or 1) in the second, and **B** is the 2 x 3 matrix of effects, with intercepts in the first row and marker effects in the second. **E** is the multivariate normal residual error. We fitted this fixed-effect model for each marker using R/qtl (Broman *et al.* 2003), calculating a p-value for the marker from an approximate F statistic based on the Pillai-Bartlett test statistic. We performed forward selection with a residual empirical threshold to build a QTL model (Doerge and Churchill 1996). After the first marker regression scan, we tested whether any marker exceeded a genome-wide empirical significance threshold of $p = 0.05$ by analyzing 1000 datasets with permuted strain labels. The top marker genome-wide that passed the empirical threshold was then incorporated into a genetic model of the trait vector, and we repeated the scan and permutations on the multivariate residuals of that model. We continued these cycles of scans and permutations until no additional markers exceeded the relevant residual empirical threshold. The final model includes eight QTL (*i.e.*, the design matrix includes nine columns), and we report the variance explained by the model for each trait as the trait-wise multiple *r*-squared from the multivariate model. We tested for epistasis among the eight QTL using approximate *F*-statistics based on the Pillai-Bartlett trace to compare an additive model and one with all pairwise interactions, and then an additive model and one with all possible interactions.

Analysis of Near-Isogenic Lines

Each strain was measured in an average of 14.8 independently passaged replicate populations (3 assay days x 5 passaging replicates each, less some missing data), and we used the Best Linear Unbiased Predictor for each of the 237 growth replicates (14.8 growth replicates x 16 strains) as the phenotype corresponding to that replicate. In the dataset of 237 BLUPs, strain identity accounts for 61% of variation in each size-class proportion and 43% of variation in number of progeny. To test for differences between pairs of strains, we performed a multivariate analysis of variance, using only the BLUPs from that pair of strains (i.e., an average of 14.8 observations for each of the two strains). We then estimated p-values by permuting the strain labels for these two-strain datasets. See File S3 for the R script that implements these analyses.

Simulations

To simulate number-of-progeny phenotypes for the RIAILs, we assigned QTL effects to 600 of the 1454 markers in the RIAIL genotype data. Effect sizes of the QTL were simulated in several ways:

1) Effects were drawn from a normal distribution with the mean and standard deviation estimated from the 15 QTL effects estimated from the NILs (i.e, including the non-significant ones). This gives a mean effect of -0.1 and a standard deviation of 8.6.

2) Effects were drawn from a uniform distribution with the same range as the 15 QTL effects estimated from the NILs. The resulting uniform distribution ranges from -12.6 to 12.4.
3) Effects were drawn from the empirical effect size distribution by resampling 600 times from the 15 estimated QTL effects.
Histograms representing these distributions are shown in figure S5.
Note that our simulations involve two assumptions that understate the excess variance implied by the NIL QTL effects. First, we include the nonsignificant (smaller) QTL in the simulated effect sizes, though we count only the significant QTL in extrapolating the number of QTL in the genome. Second, we attempt to match the RIAIL phenotypic variance, though only a fraction of that variance will be due to genetic effects.

Supplementary References

Andersen, E. C., J. S. Bloom, J. P. Gerke and L. Kruglyak (2014). A variant in the neuropeptide receptor npr-1 is a major determinant of *Caenorhabditis elegans* growth and physiology. *PLoS Genet* 10: e1004156.

Andersen, E. C., T. C. Shimko, J. R. Crissman, R. Ghosh, J. S. Bloom, H. S. Seidel*, et al.* (2015). A powerful new quantitative genetics platform, combining *Caenorhabditis elegans* high-throughput fitness assays with a large collection of recombinant strains. *G3* 5: 911-920.

Bernstein, M. R. and M. V. Rockman (2016). Fine-scale crossover rate variation on the *Caenorhabditis elegans* X chromosome. *G3* 6: 1767-1776.

Boyd, W. A., M. V. Smith and J. H. Freedman (2012). *Caenorhabditis elegans* as a model in developmental toxicology. *Methods Mol Biol* 889: 15-24.

Broman, K. W., H. Wu, S. Sen and G. A. Churchill (2003). R/qtl: QTL mapping in experimental crosses. *Bioinformatics* 19: 889-890.

Doerge, R. W. and G. A. Churchill (1996). Permutation tests for multiple loci affecting a quantitative character. *Genetics* 142: 285-294.

Knott, S. A. and C. S. Haley (2000). Multitrait least squares for quantitative trait loci detection. *Genetics* 156: 899-911.

McGrath, P. T., M. V. Rockman, M. Zimmer, H. Jang, E. Z. Macosko, L. Kruglyak*, et al.* (2009). Quantitative mapping of a digenic behavioral trait implicates globin variation in *C. elegans* sensory behaviors. *Neuron* 61: 692-699.

Sterken, M. G., L. B. Snoek, J. E. Kammenga and E. C. Andersen (2015). The laboratory domestication of *Caenorhabditis elegans*. *Trends Genet* 31: 224-231.

Stinchcombe, J. R., C. Weinig, K. D. Heath, M. T. Brock and J. Schmitt (2009). Polymorphic genes of major effect: consequences for variation, selection and evolution in *Arabidopsis thaliana*. *Genetics* 182: 911-922.

Zhao, Y., L. Long, W. Xu, R. F. Campbell, E. E. Large, J. S. Greene*, et al.* (2018). Changes to social feeding behaviors are not sufficient for fitness gains of the *Caenorhabditis elegans* N2 reference strain. *Elife* 7, e38675.

Figure S1

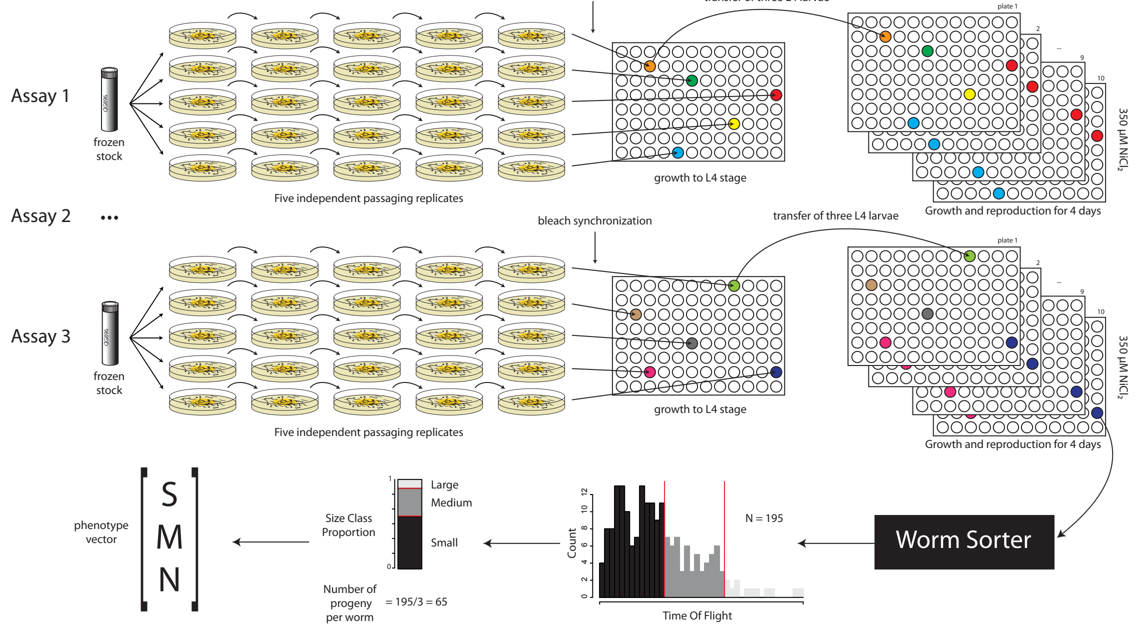

Figure S1. Experimental workflow for NILs. On each of three assay days, each NIL strain was measured from five independent passaging-replicate population. From each of these populations, we initiated populations in nine to eleven 96-well plates, each well founded by three synchronized L4 worms. Strains were assigned to random well positions. The figure shows only the five replicate populations of a single strain, but all of the strains were grown simultaneously in the 96-well plates. After four days of growth and reproduction, each well was passed through a worm sorter, which counts and measures the worms, yielding a histogram. We divided this histogram into three size classes. We then took the proportion of small worms (S), the proportion of medium-sized worms (M), and the number of progeny per initial worm (N) as the three values in a phenotype vector.

Figure S2

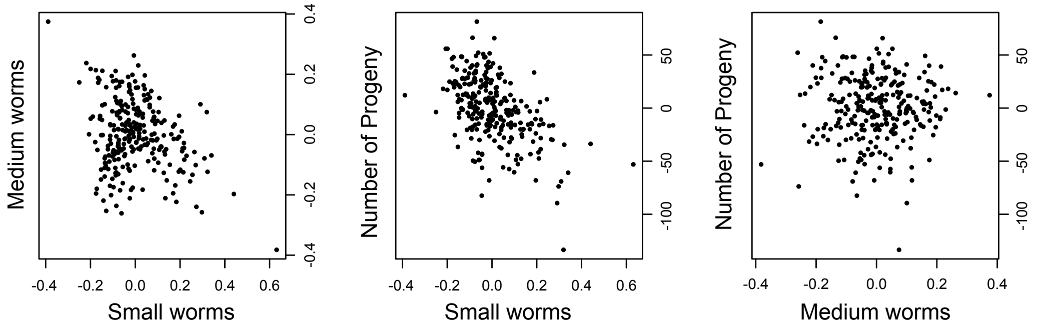

Figure S2. Assay-adjusted multivariate phenotypes for 272 RIAILs.

Figure S3

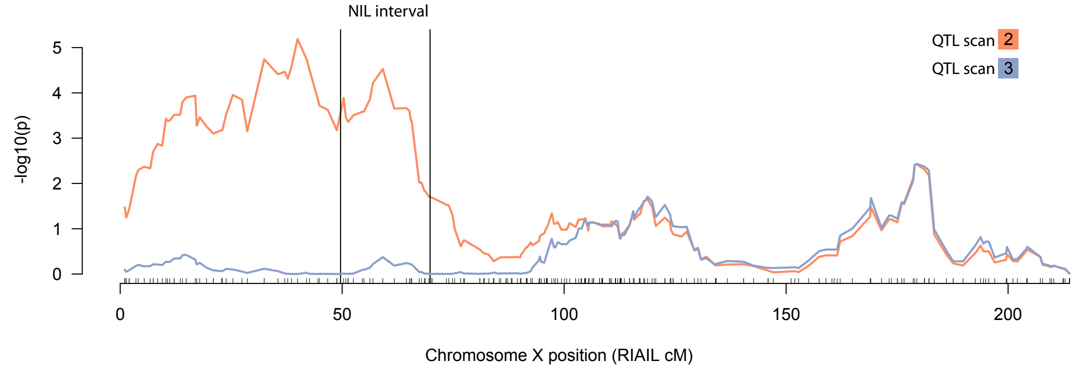

Figure S3. The NIL interval falls on the shoulder of QTL 2. When the genotype at the QTL peak marker is included as a covariate in a QTL scan (i.e, scan 3), the NIL interval does not show an effect.

Figure S4

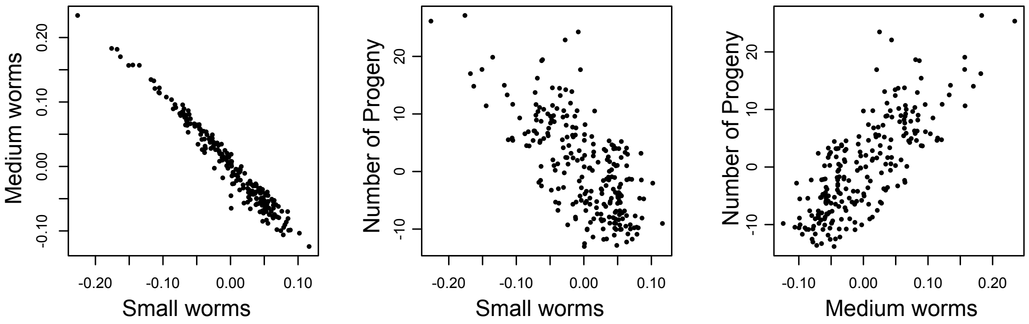

Figure S4. 237 estimated phenotypes for the 16 Near Isogenic Lines. Each point is the random-effect estimate for a set of 9-11 experimental replicates, after accounting for assay day, well position, assay plate.

Figure S5

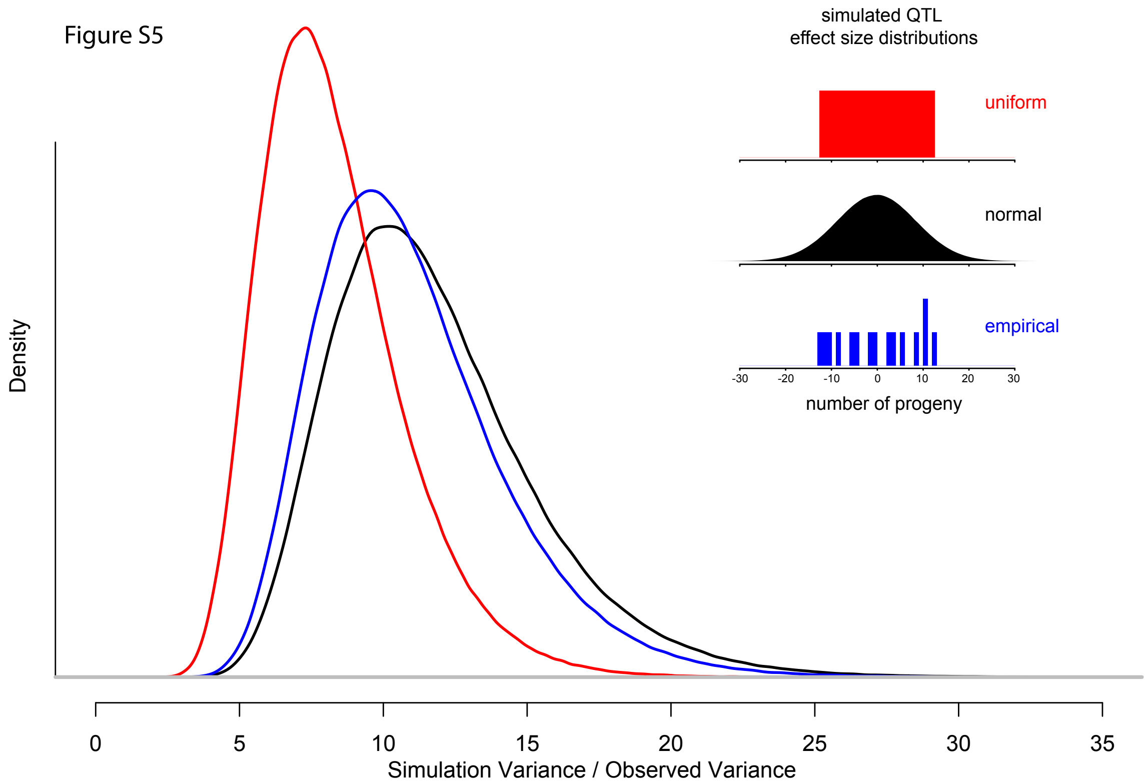

Figure S5. The NIL QTL imply excessive phenotypic variance for the RIAILs under a null model of randomly distributed QTL. These densities plot the distribution of the ratio of simulated to observed phenotypic variance, when we simulate 600 QTL with effects drawn from three different distributions.

Figure S6

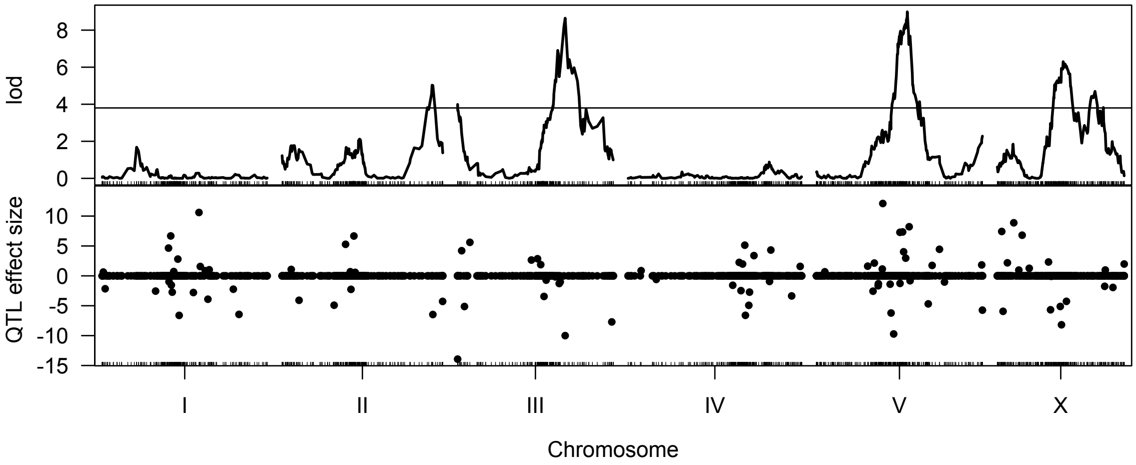

Figure S6. A representative simulation of 100 QTL with effects drawn from a normal distribution with standard deviation 4.5. The phenotypes of 272 RIAILs were simulated by assigning the QTL to 100 markers in the RIAIL genotype data, calculating the expected phenotypes, then adding an equivalent amount of random variation (i.e., expected broad sense heritability is 0.5). For this simulation, the resulting phenotypic variance was 0.98 of the variance observed in the real RIAIL data. The top panel shows a univariate QTL scan, with the genome-wide threshold set at 3.8. The QTL scan is qualitatively similar to the real results (Figure 2). The bottom panel shows the distribution of simulated QTL and their effect sizes along the genome.

Table S1: Permutation-based NIL p-values

Interval	Multivariate p	univariate p: Small worms	univariate p: Medium worms	univariate p: Number of progeny
a	0.000016	0.000930	0.000210	0.057818
b	0.002648	0.002502	0.000163	0.001005
c	0.014480	0.928243	0.922571	0.150862
d	0.163658	0.833970	0.629455	0.217539
e	0.231720	0.035791	0.039201	0.070692
f	0.000164	0.000001	0.000001	0.000101
g	0.000000	0.007993	0.000087	0.000000
h	0.000288	0.383628	0.013692	0.000121
i	0.000098	0.000085	0.000034	0.256735
j	0.038254	0.693053	0.320934	0.045185
k	0.066972	0.070473	0.147578	0.971157
l	0.000473	0.000820	0.000369	0.000051
m	0.003677	0.000585	0.000374	0.000337
n	0.000002	0.359286	0.966371	0.000394
o	0.000000	0.000000	0.000000	0.000000
Parental NILs	0.001222	0.000545	0.002090	0.796328

p-values from 1,000,000 permutations. Each test is based on a multivariate or univariate comparison of the 12-15 BLUPs for each of two NILs.
